# Remediation of Chlorinated Solvent Plumes Using *In-Situ* Air Sparging—A 2-D Laboratory Study

**DOI:** 10.3390/ijerph8062226

**Published:** 2011-06-16

**Authors:** Jeffrey A. Adams, Krishna R. Reddy, Lue Tekola

**Affiliations:** 1 ENGEO Incorporated, 2010 Crow Canyon Place, Suite 250, San Ramon, CA 94583, USA; E-Mail: jadams@engeo.com; 2 Department of Civil & Materials Engineering, University of Illinois at Chicago, 842 West Taylor Street, Chicago, IL 60607, USA; E-Mail: gageluic@gmail.com

**Keywords:** air sparging, groundwater, contamination, non-aqueous phase liquids, soils, remediation, pollution

## Abstract

*In-situ* air sparging has evolved as an innovative technique for soil and groundwater remediation impacted with volatile organic compounds (VOCs), including chlorinated solvents. These may exist as non-aqueous phase liquid (NAPL) or dissolved in groundwater. This study assessed: (1) how air injection rate affects the mass removal of dissolved phase contamination, (2) the effect of induced groundwater flow on mass removal and air distribution during air injection, and (3) the effect of initial contaminant concentration on mass removal. Dissolved-phase chlorinated solvents can be effectively removed through the use of air sparging; however, rapid initial rates of contaminant removal are followed by a protracted period of lower removal rates, or a tailing effect. As the air flow rate increases, the rate of contaminant removal also increases, especially during the initial stages of air injection. Increased air injection rates will increase the density of air channel formation, resulting in a larger interfacial mass transfer area through which the dissolved contaminant can partition into the vapor phase. In cases of groundwater flow, increased rates of air injection lessened observed downward contaminant migration effect. The air channel network and increased air saturation reduced relative hydraulic conductivity, resulting in reduced groundwater flow and subsequent downgradient contaminant migration. Finally, when a higher initial TCE concentration was present, a slightly higher mass removal rate was observed due to higher volatilization-induced concentration gradients and subsequent diffusive flux. Once concentrations are reduced, a similar tailing effect occurs.

## 1. Introduction

Air sparging has emerged as an innovative technique for soil and groundwater remediation [1–3]. In a typical field system, compressed air is transported through a manifold system which, in turn, delivers air to an array of air injection wells. The well sparge point is located below the lowest known point of contamination. Due to buoyancy, the injected air rises towards the ground surface, and through variety of mass transfer, transport, and transformation mechanisms, the contamination present within the subsurface partitions into the vapor phase or is degraded. As the contaminant-laden air continues to rise toward the subsurface it encounters the vadose zone of soil, where it is often captured using a soil vapor extraction (SVE) system. By applying a vacuum to the subsurface, the SVE system is also able to control the movement of vapors; encouraging movement toward the extraction wells while preventing unwanted migration into soil areas previously unaffected by contamination. Once extracted, the contaminated air may be treated using conventional methods such as carbon filters or combustion. If the conditions are favorable within the vadose zone, the native subsurface microbial population may degrade the contamination into harmless products.

Volatile organic compounds (VOCs) released into saturated subsurface commonly exist in free-phase as non-aqueous phase liquid (NAPL) or dissolve into groundwater. VOCs that commonly contaminate groundwater include petroleum hydrocarbons and chlorinated solvents. Because of their ideal and unique properties, chlorinated solvents such as trichloroethylene (TCE) have been widely used in industrial cleaning solutions and as “universal” degreasing agents and are frequently detected within groundwater in United States. These compounds often have low water solubility but high vapor pressure. The latter property makes them easily amenable to techniques like air sparging and soil vapor extraction. Since these compounds have low water solubility, a significant NAPL phase fraction will often exist within the subsurface.

Many studies have been performed to assess air sparging, involving either the evaluation of field studies, physical models, or mathematical models [4–10]. A number of previous studies have focused upon NAPL transport within the subsurface due to both gravity-induced infiltration and groundwater flow. Previously conducted air sparging studies have focused on LNAPLs (often benzene) as the model VOC [11]. An investigation into removal of free-phase DNAPL (TCE) by air sparging was investigated previously [12,13]. The purpose of this study is to investigate the removal of dissolved DNAPL plumes such as TCE plume from groundwater using *in-situ* air sparging. Particularly, this paper investigates the effects of air injection rate, groundwater flow and initial dissolved TCE concentrations on the TCE mass removal.

## 2. Experimental Program

### 2.1. Aquifer Simulation Setup

**Figure 1.**
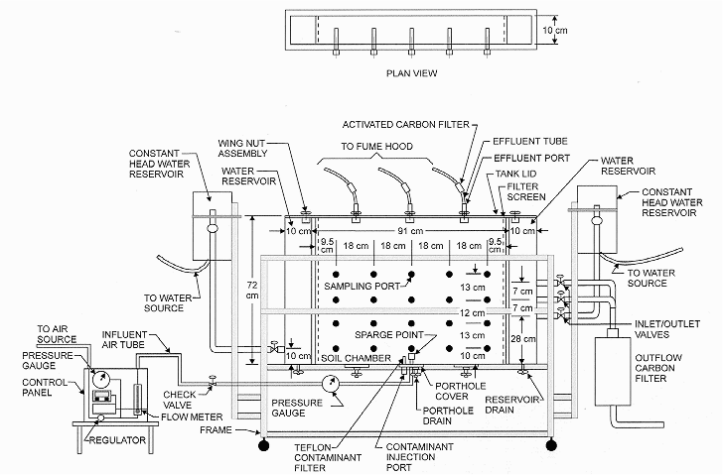
Schematic of two-dimensional aquifer test setup.

A two-dimensional aquifer simulation test apparatus, shown in Figure 1, was used in this study. It is made of Plexiglas, measuring 111 cm in length, 72 cm in height, and 10 cm in width. The interior of the apparatus consists of three compartments; a central soil chamber measuring 91 cm in length is flanked by two groundwater reservoir chambers, measuring 10 cm in length each. The reservoir chambers are supplied by two constant-head clean water reservoirs. These reservoirs are adjustable in height, allowing for a specific head to be maintained in each reservoir. Additionally, the source reservoirs may be adjusted to create a hydraulic gradient across the apparatus, thereby inducing groundwater flow. The apparatus reservoirs are separated from the soil chamber by geotextile-lined heavy-gauge perforated stainless steel screen, allowing water to freely enter or exit the central soil chamber but preventing soil particles from entering the water reservoirs. One face of the apparatus includes twenty sampling ports arranged in an array of four rows and five columns. The ports, which extend into the central soil chamber, allow for pore water sampling from within the soil matrix. The ports allow sampling of pore water from the soil within the soil chamber (“soil profile”) throughout the course of the testing. The bottom of soil chamber consists of air injection port. The setup cover makes it completely air tight and the effluent gas outlets are fitted with filters. All other peripherals of the test setup are shown in Figure 1. More details on the setup can be found in Adams [11] and Tekola [12].

### 2.2. Materials

For this investigation, tricholoroethylene (TCE) was used as a representative contaminant given its status as a common chlorinated solvent contaminant within soil and groundwater. Uniform coarse sand obtained from U.S. Silica Company (U.S Silica designation 20/40 fraction) was used as the test soil for this research. The sand is poorly graded, with a D_10_ = 0.43 mm and a D_50_ = 0.55 mm. The hydraulic conductivity of sand is 4.64 × 10^−2^ cm/sec and the porosity under test conditions had a value 0.4. This clean sand, free of organic content, minimized the potential of TCE soil matrix adsorption.

### 2.3. Testing Program

All the tests were performed using soil profiles constituted of homogeneous coarse sand. Six different tests were performed; the first three tests were conducted under different air injection rates under static groundwater condition. The next two tests were conducted under two different air injection rates, but under simulation of flowing groundwater conditions. All of these tests were conducted using the soil profiles with dissolved TCE concentration of 300 mg/L. The final test was conducted with a higher dissolved concentration of 500 mg/L to investigate the effect of initial concentration on mass removal. The selected air injection rates for the tests were: 2,225 mL/min, 4,000 mL/min, and 7,156 mL/min and were injected under air pressure of 6.9 kPa. When considering reported field injection rates between 100 and 500 liters per minute, these injection rates are appropriate representations considering the scaling effects of the bench-scale testing apparatus.

### 2.4. Testing Procedure

A 300-mg/L TCE solution was prepared using a 4-liter capacity wide-mouth borosilicate glass bottle with a modified lid. The lid was fitted with a septum, allowing for injection of TCE into the bottle while minimizing mass loss through volatilization. A magnetic stirring bar was placed into the bottle, and the bottle was then filled with 3,735 mL of de-ionized water, eliminating head space. Pure-phase TCE was injected into the water through the septum to create a solution with the desired TCE concentration. The solution was completely mixed overnight.

In preparing the soil matrix for testing, the soil was placed into the apparatus from the top at a controlled rate with constant drop height, creating a uniform soil profile. Once the soil was placed, a peristaltic pump was used to inject the TCE solution into the dry soil through a port located on the bottom of the testing apparatus, until approximately three-fourths of the soil profile had been saturated with the TCE solution. The constant-head clean water reservoirs were also connected to the system, saturating areas outside of the contaminated zone with clean water and maintaining a constant head condition during air injection.

Once the soil profile had been saturated, pore water was sampled from each port and analyzed to yield the initial concentration profile prior to air injection. Although contaminant mass loss during injection but prior to air injection via volatilization may have occurred, the losses were minor (as demonstrated by the initial concentration profiles). Further, lost mass would have been captured in the ORBO tubes attached to the effluent lines, as described below. Pore water was also sampled during the course of each test at regularly scheduled time intervals. Gas tight syringes were used to extract 100 μL pore water samples from each port. Extracted pore water was analyzed using a gas chromatograph (GC) to determine TCE concentrations [11,12].

During air injection, the effluent gas exited the test apparatus and passed through ORBO activated carbon filter tubes. The ORBO tubes were used to trap the TCE removed from the soil profile, thereby allowing for a mass balance analysis. Multiple ORBO tubes were used in series to avoid contaminant breakthrough. Additionally, the ORBO tubes were changed at regular intervals to monitor the mass of contaminant removed during a specific time interval. Two tube sizes were used: 400/200 mg large tubes and 800/200 mg jumbo tubes. The actual number of tubes used at any given time was dependent on the elapsed time of the test; more tubes were used at the early stage because of higher contaminant removal during the initial stages of air injection. Following removal from the effluent lines, the tubes were sealed and refrigerated until extraction analyses was performed.

## 3. Results and Analysis

### 3.1. Effect of Air Flow Rate under Static Groundwater Condition

The first three tests included a static groundwater condition and an initial TCE concentration of 300 mg/L. In all cases, the pore water sampling performed prior to air injection identified a concentration gradient within the contaminated soil profile. Higher initial TCE concentrations were observed in the bottom of the soil profile as compared to those measured at the top of the soil profile [12]. The gradient is attributed to the injection process; the contaminant solution was injected at the bottom of the apparatus, which induced a wetting front and migration toward the top of the soil profile. The natural retardation factor of dissolved TCE within migrating groundwater likely contributed to the resulting gradient. Some adsorption to the soil media may also have occurred; however, this is expected to have been minimal due to the use of silica sand.

**Figure 2.**
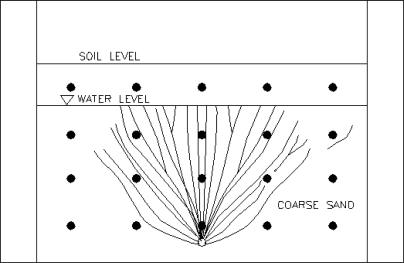
Zone of influence and air flow distribution under air flow rate of 2,225 mL/min.

During the first test, air was injected at a rate of 2,225 mL/min (medium flow rate) under a pressure of 6.9 kPa. A dense network of air channels formed near the sparge point. The air channels decreased in density with increased vertical and lateral distance from the sparge point. As the air injection commenced, a relatively high air channel density was observed within the initial contaminant zone. A schematic of a representative zone of influence (ZOI) is shown in Figure 2. However, the channel density was much lower with increased lateral distance from the sparge point, outside of the initial contaminant zone.

**Figure 3.**
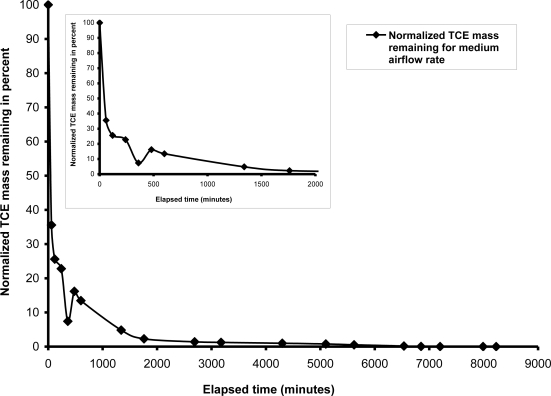
TCE mass removal with air flow rate = 2,225 mL/min and static groundwater condition.

A rapid decrease in TCE concentration was observed during the first four hours of air injection and 50 percent of the TCE concentration reduction was observed during the first 30 minutes of air injection. Figure 3 shows the rapid TCE mass and concentration reduction was followed by a characteristic “tailing” effect in which the rate of subsequent concentration reductions decreased noticeably. Approximately 90 percent of the TCE mass had been removed within the first 1,000 minutes, but residual concentrations were still present after 7,000 minutes of injection. Most of the residual TCE concentrations were located outside of the primary zone of influence (ZOI) after 4,300 minutes. The rapid TCE concentration reductions that occurred during the initial stages of air injection are attributable to the dominance of volatilization as a mass transfer process. A concentration gradient was induced between areas within the ZOI treatment zone (low concentrations of TCE) and areas outside of the ZOI (higher TCE concentrations). The concentration gradient induced diffusive transport of the TCE from the higher concentration areas into lower concentration areas, resulting in eventual removal upon migration into areas with greater air channel density within the ZOI. Following the rapid TCE concentration reduction during the early injection period, diffusion became a rate-limiting transport mechanism, resulting in the observed tailing effect.

**Figure 4.**
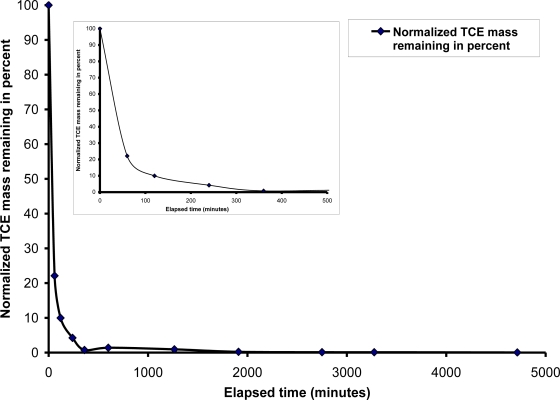
TCE mass removal with air flow rate = 4,000 mL/min and static groundwater condition.

For the second test, air was injected at a rate of 4,000 mL/min (high air flow) under a pressure of 6.9 kPa and with a static groundwater condition. Upon commencement of air injection, the ZOI was very similar to that observed in the medium air flow test, except a higher density of the channels was observed. Significant TCE concentration reductions were observed during the initial stages of air injection (Figure 4). The rate of removal decreased with increased injection time, with the exception of areas adjacent to the sparge point or outside of the ZOI. Eventually, concentration reductions were observed at later stages of injection, including the top left of the contamination zone. Overall, the pattern of contaminant removal was similar to that observed during 2,225 mL/min air injection flow. There was a higher rate of contaminant removal during the early stages of the air injection; 90 percent of the normalized TCE mass was removed within 3 hours (Figure 4). As testing progressed mass removal decreased, exhibiting a similar tailing effect, indicating that rate-limiting mass transfer processes, especially diffusion, had become dominant. Nevertheless, this effect was less pronounced and the residual concentrations were consistently lower using a high flow rate compared to a medium flow rate. The higher air injection rate induced a greater rate of volatilization, especially during the first few hours of air injection. This is clearly demonstrated by comparing the concentration profile of the medium air flow test with high air injection flow test for the same duration of air injection. However, as in the case of the medium air flow test, the period of rapid concentration removal was followed by a tailing effect in which concentration reductions occurred slowly. Furthermore, many zones outside of the ZOI exhibited residual TCE concentrations, even after 2,000 minutes of air injection.

**Figure 5.**
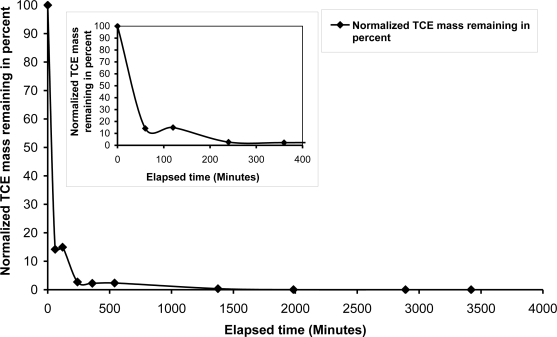
TCE mass removal with air flow rate = 7,156 mL/min and static groundwater condition.

The third test was performed with a static groundwater condition, this time with an air injection rate of 7,156 mL/min (very high air flow) under a pressure of 6.9 kPa. An increased density of air channels were formed within and outside the initial contaminant zone as compared to those with lower flow rates. The rate of TCE removal was faster than that observed during the high air flow rate test. Almost 90 percent of the initial TCE mass was removed within the first hour of air injection (Figure 5). As expected, a high TCE removal rate was observed in regions of high air channel density.

As demonstrated during these tests, volatilization was the dominant contaminant transport mechanism. Dissolved TCE within pore water was easily removed from the contaminated profile in areas adjacent to a dense air channel network. However, with increased air flow rates, pore-scale agitation enhanced mechanical dispersion, which in turn enhanced the partitioning of trapped dissolved TCE into the vapor phase. Additionally, the concentration gradient that developed between areas within and outside of the ZOI induced diffusive transport of TCE from these outside areas into the ZOI. This diffusion process was slow, as evidenced by the slower rates of concentration reductions as compared to the rates observed within the ZOI. This indicates that diffusion is a rate limiting process for remediation in areas outside of a sparge point ZOI.

### 3.2. Effect of Air Flow Rate under Groundwater Flow Condition

The next series of tests were performed with an induced groundwater flow. Following the initial saturation of the soil profile with the TCE solution and clean water, the constant-head clean water reservoirs were adjusted in height to induce groundwater flow under a hydraulic gradient of 0.011. Once groundwater flow was established across the soil profile, air injection commenced.

**Figure 6.**
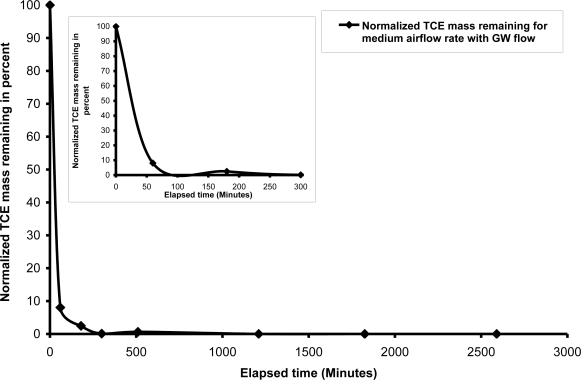
TCE mass removal with air flow rate = 2,225 mL/min and dynamic groundwater flow condition.

For the first test, air was injected at a rate of 2,225 mL/min (high air flow) under a pressure of 6.9 kPa. Once the air injection began, the groundwater gradient and flow was disrupted; therefore, the constant-head reservoirs were adjusted to maintain the desired flow and gradient. The resulting air flow pattern and ZOI were similar to previous tests performed with a static groundwater condition; with the exception of a decreased air channel density in the upper left (“upgradient”) region of the initial contaminant zone. However, it is unclear if this was the result of the induced groundwater flow. Nevertheless, the introduction of injected air did reduce relative hydraulic conductivity of the soil profile, as evidenced by the disrupted groundwater flow. Although the initial contaminant distribution was mainly restricted to the initial contaminant zone, the hydraulic gradient induced a TCE concentration gradient across the soil profile from left to right. TCE concentrations increased in the downgradient direction, having been induced by groundwater flow, even prior to the start of air injection. As in the case of the static groundwater condition, significant contaminant removal was observed in the first 60 minutes of air injection, especially near the sparge point. This rapid mass removal is attributed to both volatilization and advection/dispersion induced by groundwater flow. Complete mass removal occurred within approximately 1,200 minutes (Figure 6).

**Figure 7.**
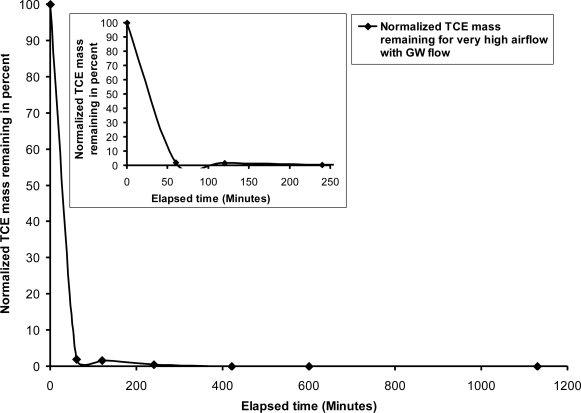
TCE mass removal with air flow rate = 7,156 mL/min and dynamic groundwater flow condition.

The observed rate of removal was quite similar in most locations of the soil profile [12]. As observed in tests with a static groundwater condition, rapid rates of TCE concentration reductions within the first hour of injection were followed by a tailing effect, resulting in eventual removal. When the air channel network formed, volatilization induced rapid mass reductions; however, as was observed with a static groundwater condition, the tailing effect was caused by the rate-limiting effect of diffusion. Dissolved TCE trapped within dead-end pore space was reliant upon diffusion for removal. Conversely, and perhaps counter-intuitively, faster mass reductions occurred in the upper levels of the soil profile, corresponding to areas with a lower air channel density. This is perhaps attributable to the greater effect of groundwater flow (due to a lessened reduction in hydraulic conductivity from air injection) and a greater degree of advection/dispersion. Groundwater effluent was also monitored. Trace concentrations were detected in effluent prior to air injection. However, during the initial stages of air injection, effluent TCE concentrations increased due to the induced advection/dispersion caused by the injected air. For instance, following 60 minutes of air injection, an effluent concentration of 248 mg/L was detected. Subsequent sampling indicated a decrease in effluent concentrations. The effluent did not exhibit detectable TCE concentrations after 30 hours of injection. An additional test was performed with induced groundwater flow (hydraulic gradient of 0.011) and an air injection rate of 7,156 mL/min (very high air flow) under a pressure of 6.9 kPa. A similar air channel pattern developed as compared to the previous test; however, as observed with a static groundwater condition, the zone immediately above and near the sparge point exhibited a higher degree of air channel formation. The greater channel density created a greater interfacial mass transfer area to induce volatilization, but it also led to a greater reduction in hydraulic conductivity and groundwater flow. As in the case of the test with 2,225 mL/min air flow, the rate of TCE removal was greater during the early stages of the air injection. This relatively higher rate of removal occurred in the first hour of the air injection, followed by a tailing effect. Complete mass removal occurred within 410 minutes of air injection (Figure 7). Regions near the sparge point experienced rapid concentration reductions, followed by a pronounced tailing effect. Even though the greater degree of air channel density led to higher volatilization rates, as the TCE concentration declined, total mass removal became more dependent on rate-limiting diffusion. Additionally, the greater air channel density further decreased relative hydraulic conductivity, reducing the effects of groundwater flow-induced advection/dispersion.

Although regions downgradient from the ZOI initially free of contamination experienced infiltration due to groundwater-induced advective-dispersive transport, the extent was lessened as compared to that observed in the medium air flow test due to the reduced hydraulic conductivity and resulting groundwater flow. Also, as in the case of the medium air flow test, the groundwater effluent exhibited trace TCE concentrations prior to air injection, but greater concentrations during air injection. The peak TCE concentration was detected after one hour of air injection similar to that observed during medium air injection flow rate test. However, this effect was lessened as compared to the test with medium air flow. Although the decrease in relative hydraulic permeability of the porous media caused by higher air saturation could not stop downgradient contaminant migration, the effects were less pronounced, indicating the increased air saturation prevented downgradient migration to an appreciable extent. Therefore, increased air injection results in two important phenomena. First, increased air injection creates an increased air channel density and the mass transfer area necessary to induce volatilization. Secondly, increased air injection and the resulting air channel density reduced relative hydraulic conductivity within the ZOI. This reduced downgradient contaminant migration, which increased the contaminated groundwater residence time within the ZOI. Additionally, the reduced hydraulic conductivity and increased residence time lessened the degree of contaminant transport, or “smearing”, into downgradient regions unaffected by air flow, thus confining contamination to a region where more efficient removal occurred.

### 3.3. Effect of Higher Initial Dissolved Concentration

**Figure 8.**
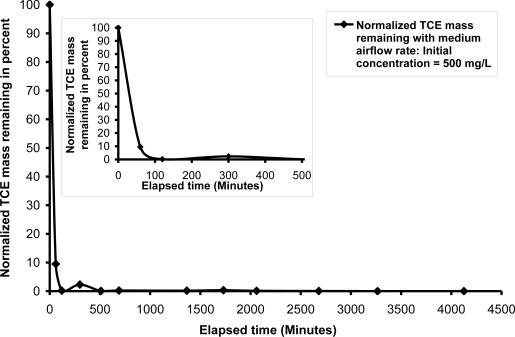
Effect of higher initial dissolved TCE concentration on mass removal with air flow rate = 2,225 mL/min and dynamic groundwater flow condition.

An additional test was performed to study the effect of the initial concentration of the contaminant on the overall mass removal during air injection. An initial TCE concentration of 500 mg/L was used, higher than the 300 mg/L used in earlier tests performed with groundwater flow. Once again, a hydraulic gradient of 0.011 was created to induce groundwater flow. Air was injected at a rate of 2,225 mL/min under a pressure of 6.9 kPa. As shown in Figure 8, a slightly higher mass removal rate was observed as compared to previous tests, which is attributable to higher volatilization-induced concentration gradients and subsequent diffusive flux. As the test proceeded, and concentrations were reduced, the gradients approached those seen in earlier tests and a similar tailing effect occurred. This test required twice the air injection time as that observed in a similar test with a 300 mg/L initial TCE concentration. The greater TCE concentrations trapped in dead-end pores may have led to a temporary concentration increase once mobilized from the pores and prior to volatilization. As in the cases of other tests with groundwater flow, the groundwater effluent exhibited trace TCE concentrations prior to and at the beginning of air injection. This was followed by peak effluent concentrations one hour into the air injection process, which in turn was succeeded by decreased effluent concentrations as the air injection progressed. As in the other test cases, the initial increases in effluent concentrations were induced by the advection/dispersion resulting from air injection. Both the initial concentration and air injection rate have a direct impact on the TCE removal rate. However, comparatively speaking, the air injection rate has a greater impact on the removal rate than initial contaminant concentration. The test performed with the very high air flow rate has a similar initial rate of removal as that observed during the medium flow rate test, but a lessened tailing effect in the test with the very high flow rate resulting in a quicker complete mass removal time.

## 4. Practical Implications

This study has shown that air sparging can be used to remediate dissolved phase TCE contamination. In the case of dissolved phase contamination, volatilization effects are mostly limited to early stages of air injection, resulting to high initial reductions in contaminant concentration and mass. In later stages, diffusion becomes a rate-limiting mass transport process, leading to a tailing effect.

This particular study has demonstrated that special care should be exercised when dissolved phase contamination is treated using air sparging. Rate-limiting mass transfer mechanisms that occur in later stages (resulting in the observed tailing effect) lead to prolonged operation and increased operational cost. Therefore, auxiliary enhancements or other complementary remediation techniques may be coupled with air sparging operations to overcome these rate-limiting processes.

The result of this series of experiments has numerous implications for field applications of air sparging. In cases of high groundwater flow and downgradient contaminant plume migration, the air channel network and increased air saturation induced by air sparging reduces relative hydraulic conductivity, resulting in reduced groundwater flow and subsequent downgradient contaminant migration. Using an increased air injection rate may further reduce hydraulic conductivity; however; if the air injection is too high, the relative hydraulic conductivity may reduce to the extent that groundwater flow may bypass the ZOI, resulting in unintended contaminant offsite migration. Therefore, caution must be exercised in deciding the air flow rate depending on the groundwater flow conditions.

If the air injection occurs in a controlled manner and does not induce undesirable preferential groundwater flow, air sparging may be used to effectively intercept and treat migrating contaminant plumes. Additionally, care must be exercised to assure that the entire contaminant plume is intercepted to prevent contaminant from circumventing the treatment zone. Moreover, depending on the initial concentration of the contaminant plume, varying some of the variables like air injection rate will likely have significant effect on the expected remediation timeframe. This was clearly demonstrated during this series of tests, as variable injection rates, groundwater flow conditions, and initial contaminant concentrations had a pronounced effect on contaminant removal rates and total remediation time. The increased dissolved oxygen during air sparging has potential to enhance aerobic biodegradation of residual contamination, which is being researched [14,15].

## 5. Conclusions

This investigation was performed to study the removal of dissolved TCE using air sparging. The tests were performed under both static groundwater and induced groundwater conditions, using a variety of air injection rates and initial TCE concentrations. The following conclusions may be drawn from this study:

1. Dissolved phase chlorinated solvents can be effectively removed through the use of air sparging. The application of air sparging will induce partitioning of the dissolved-phase contaminant into the vapor phase via volatilization, allowing for removal from the subsurface. Additionally, the rapid initial rates of contaminant removal are followed by a protracted period of lower removal rates, or a tailing effect. As a result, the total time necessary for removal can be lengthy.
2. As the air flow rate increases, the rate of contaminant removal also increases, especially during the initial stages of air injection. Increased air injection rates will increase the density of air channel formation, resulting in a larger interfacial mass transfer area through which the dissolved contaminant can partition into the vapor phase.
3. In cases of groundwater flow, downgradient contaminant plume migration was observed; however, increased rates of air injection lessened the observed migration effect. The air channel network and increased air saturation reduces relative hydraulic conductivity, resulting in reduced groundwater flow and subsequent downgradient contaminant migration. Increased air injection rates enhanced this effect; however, if injection rates are too high, the resulting decrease in hydraulic conductivity may cause groundwater flow to bypass the ZOI, resulting in unintended contaminant offsite migration.
4. When a higher initial TCE concentration was present, a slightly higher mass removal rate was observed. This is attributable to higher volatilization-induced concentration gradients and subsequent diffusive flux. However, as the test proceeded, and concentrations were reduced, the gradients approached those seen in earlier tests and a similar tailing effect occurred. Because of the higher concentrations present, the tailing effect was more pronounced than that observed in tests with lower concentrations.

## References

[1] Reddy KR, Kosgi S, Zhou J (1995). A review of *in-situ* air sparging for the remediation of VOC-contaminated saturated soils and groundwater. Hazard. Waste Hazard. Mater.

[2] Johnson RL, Johnson PC, McWhorter DB, Hinchee RE, Goodman I (1993). An overview of insitu air sparging. Ground Water Monit. Rev.

[3] Marley MC, Hazebrouck DJ, Walsh MT (1992). The application of *in-situ* air sparging as an innovative soils and groundwater remediation technology. Ground Water Monit. Rev.

[4] Reddy KR, Adams JA (1999). Laboratory study of air sparging of TCE contaminated saturated soils and ground water. Ground Water Monit. Remediat.

[5] Ji W, Dahmani A, Ahlfeld DP, Lin JD, Hill E (1993). Laboratory study of air sparging: Air flow visualization. Ground Water Monit. Rev.

[6] Gordon MJ (1998). Case history of a large-scale air sparging/soil vapor extraction system for the remediation of chlorinated volatile organic compounds in groundwater. Ground Water Monit. Remediat.

[7] McCray JE, Tick G, Jawitz JJ, Annable M, Brusseau ML, Falta R, Gierke J, Knox R, Sabatini D, Wood AL (2010). Remediation of NAPL source zones: Lessons learned from field studies at Hill and Dover AFB. Ground Water.

[8] McCray JE, Falta RW (1997). Numerical simulation of air sparging for remediation of NAPL contamination. Ground Water.

[9] McCray JE (2000). Mathematical modeling of air sparging for subsurface remediation: State of the art. J. Hazard. Mater.

[10] Rabideau AJ, Blayden JM (1998). Analytical model for contaminant mass removal by air sparging. Ground Water Monit. Remediat.

[11] Adams JA System Effects on the Remediation of Contaminated Saturated Soils and Groundwater Using Air Sparging.

[12] Tekola L Remediation of Subsurface NAPL Contamination by *In-Situ* Air Sparging.

[13] Reddy KR, Tekola L (2004). Remediation of DNAPL source zones in groundwater using air sparging. Land Contam. Reclam.

[14] Matsumiya A, Kubota K, Kubo M (2010). Evaluating effects of air sparging for *in-situ* bioremediation. J. Soc. Mater. Sci. Jpn.

[15] Aelion CM, Kirtland BC (2000). Physical *versus* biological hydrocarbon removal during air sparging and soil vapor extraction. Environ. Sci. Technol.

